# High-fat diet-induced obesity causes an inflammatory microenvironment in the kidneys of aging Long-Evans rats

**DOI:** 10.1186/s12950-019-0219-x

**Published:** 2019-06-25

**Authors:** Thea Laurentius, Ute Raffetseder, Claudia Fellner, Robert Kob, Mahtab Nourbakhsh, Jürgen Floege, Thomas Bertsch, Leo Cornelius Bollheimer, Tammo Ostendorf

**Affiliations:** 1 Department of Geriatric Medicine, RWTH University Hospital, Pauwelsstrasse 30, 52074 Aachen, Germany; 2 Department of Nephrology and Clinical Immunology, RWTH University Hospital, Pauwelsstrasse 30, 52074 Aachen, Germany; 3 Institute of Radiology, University Hospital Regensburg, Regensburg, Germany; 4 Institute for Biomedicine of Aging, Friedrich-Alexander-Universität Erlangen-Nürnberg, General Hospital Nuremberg, Paracelsus Medical University, Nuremberg, Germany; 5 Institute of Clinical Chemistry, Laboratory Medicine and Transfusion Medicine, General Hospital Nuremberg, Paracelsus Medical University, Nuremberg, Germany; 6 Department of Geriatric Medicine, RWTH University Hospital, Pauwelsstrasse 30, 52057 Aachen, Germany

**Keywords:** Rat model, High-fat-diet, Obesity, Kidney, Inflammation

## Abstract

**Background:**

Obesity is a risk factor for chronic kidney disease (CKD). While the exact mechanisms remain unclear, inflammation may be a consequence of obesity that directly impacts the kidneys. The aim of this study was to examine the inflammatory status of the kidneys and potential ongoing renal damage, i.e., tubular damage and fibrosis after long-term obesity maintained through persistent consumption of a high-fat diet (HFD).

**Results:**

Twenty-four-week-old male Long-Evans (LEV) rats were continuously fed a control diet (CD) or HFD for 51 weeks. The mean body weight was higher in HFD-fed rats than in control diet-fed rats and markedly elevated during the last 24 weeks. Blood analyses revealed no substantial alterations in renal functional parameters by HFD consumption but a substantial increase in creatine kinase, a muscle loss marker. Magnetic resonance imaging (MRI) was utilized to quantify rat quadriceps muscle mass. The data showed that HFD-induced obesity in LEV rats was accompanied by minor decreases in muscle mass and strength at 75 weeks of age. Rat kidney inflammatory status was evaluated using histological and immunohistological techniques. The number of foci with immune cell infiltrates and infiltrating monocytes/macrophages was significantly increased in HFD-fed rat kidneys at week 75. Renal fibrosis parameters, including glomerulosclerosis and tubular damage, were also markedly increased in renal tissues from HFD-fed rats compared to the controls. The significant increase in tubular protein casts in HFD-fed rat tissues indicated that renal function was already disturbed. Rat kidney inflammatory status was further evaluated using the simultaneous profiling of twenty-two inflammatory markers in kidney tissue extracts. Consistently, MCP-1 and eotaxin (CCL11) levels were elevated in obese LEV rat kidneys.

**Conclusions:**

Compared to CD-fed rats, HFD-fed obese LEV rats show significant damage of renal structures with aging. These subtle changes may sensitize the kidneys to the development of progressive CKD.

**Electronic supplementary material:**

The online version of this article (10.1186/s12950-019-0219-x) contains supplementary material, which is available to authorized users.

## Background

Obesity is an important health threat that is associated with cardiovascular morbidity and loss of skeletal muscle, known as sarcopenia. Clinical studies have also suggested a possible link between measures of obesity and both the development and progression of chronic kidney disease (CKD) [1, 2]. CKD is the common endpoint of most renal diseases and has emerged as a worldwide public health issue [3]. The underlying pathologic process of CKD is renal fibrosis associated with unremitting renal inflammation. Renal damage and inflammation often start in the glomeruli and then spread to the tubulointerstitium [4]. The suggested mechanisms causing this spreading include proinflammatory cytokines from inflamed glomeruli that might perfuse tubulointerstitial capillaries and cause inflammation or abnormal amounts of protein reabsorbed from the glomerular filtrate, potentially inducing stress responses in tubular epithelial cells [5]. The resulting inflammatory processes are characterized by tubulointerstitial mononuclear cell infiltrates contributing to immunopathology and progressive tissue remodeling, such as the infiltration of activated T cells that produce proinflammatory cytokines [6]. As a result, tubular atrophy, accumulation of interstitial myofibroblasts and development of interstitial scarring lead to the replacement of functional nephrons by fibrotic tissue [7].

In general, the associations between obesity and impaired renal outcomes persist even after adjustments for possible cardiovascular and metabolic effects, suggesting that obesity independently affects kidney function [8]. Notably, controlled animal studies revealed that a few weeks of rapid weight gain leads to structural changes in the kidneys, including enlargement of Bowman’s space, increased glomerular cell proliferation, thicker mesangial matrix and basement membranes [9]. These early renal changes occurred with no evidence for initial renal malfunction; however, if progressive, they could eventually impact the glomerular lumen, reduce the filtration surface area and lead to kidney injury. This slowly developing vicious cycle may be accelerated in the setting of metabolic derangements induced by obesity, such as inflammation or oxidative stress.

Both the extent and dynamics of tissue inflammation are highly regulated by chemokines, a family of approximately 50 small cytokines that induce directed chemotaxis in responsive cells [10]. In the kidney, the chemokine MCP-1 (CCL2) was shown to be an important player in renal inflammation that drives macrophage tissue infiltration by binding to its receptor CCR2, expressed mainly on monocytes and macrophages [11, 12]. Eotaxin (CCL11) is a potent eosinophil chemokine; however, little is known about its role in renal inflammation. Earlier studies reported that the specific expression of eotaxin in human kidney tissue may play a crucial role in renal interstitial eosinophilia [13].

The aim of our study was to investigate the effects of a long-term high-fat diet (HFD) on renal inflammation in a rat model that accurately reflects the conditions leading to the current epidemic of human obesity resulting from the industrialization of food systems. Here, we show the HFD-induced inflammatory profile of the kidneys from male Long Evans (LEV) rats, representing a model of obesity [14–16].

## Results

### Long-term HFD leads to obesity and impairment of muscle function in aging rats

**Fig. 1:**
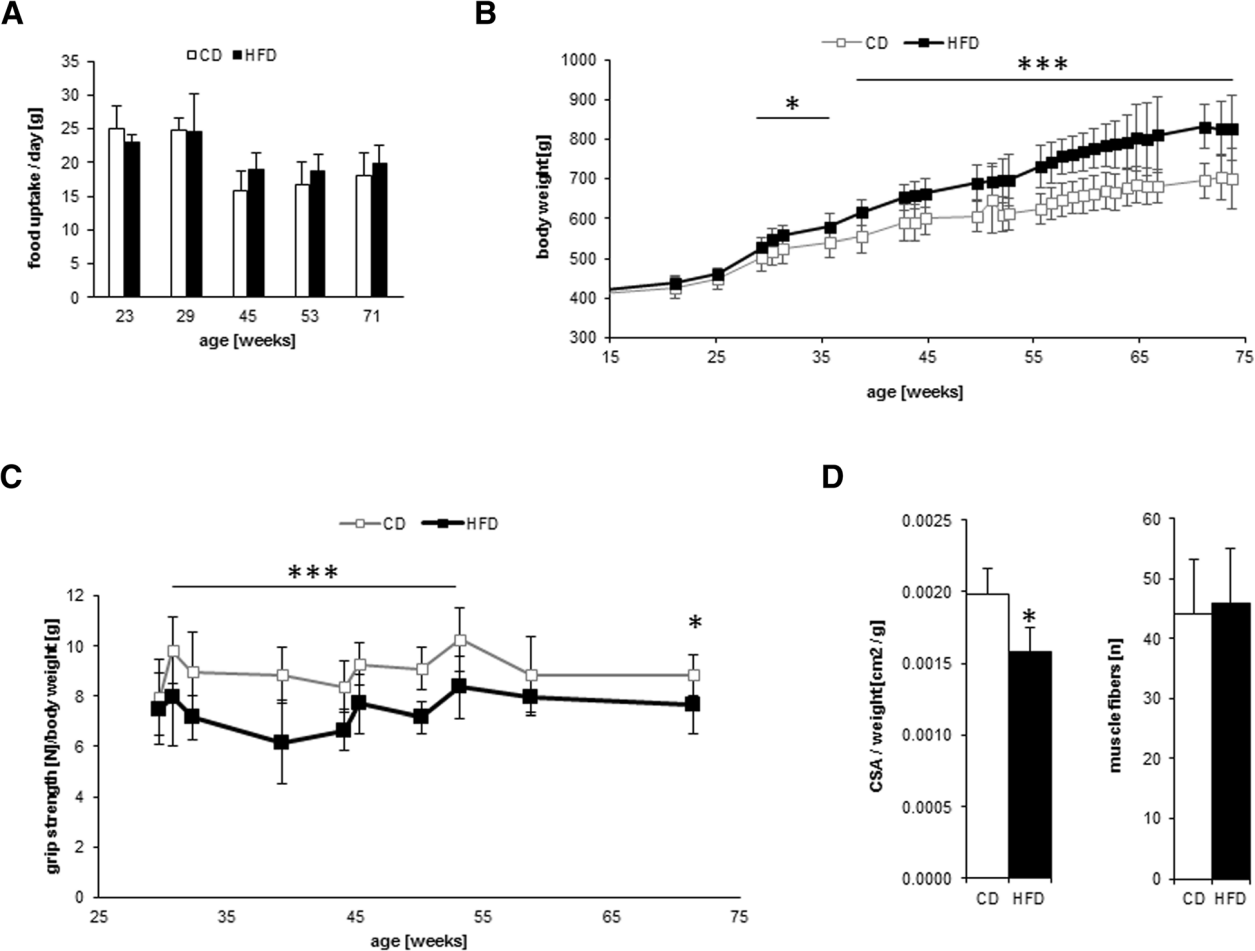
Characteristics of male LEV rats in the study. Six-month-old LEV rats were divided into two groups of 8 animals each. One group was fed a control diet (CD) (solid white bars or squares) and the other group was fed a high-fat diet (HFD (solid black bars or squares) continuously. (**a**) Mean food uptake of LEV rats in the course of the study. (**b**) Weight increase of LEV rats in the course of the study. (**c**) Mean grip strength of 18-month-old LEV rats. (**d**) The mean number of 200 ± 10% fibers was measured using HE-stained sections of the *musculus vastus lateralis* in 18-month-old LEV rats (right panel). The relative mean cross-sectional area (CSA) of the *musculus vastus lateralis* compared to body weight (left panel). Statistical significance (HFD vs. CD, at each time point) was determined using two-way t-tests and is denoted by * for *p* ≤ 0.05, ** for *p* ≤ 0.01 or *** for *p* ≤ 0.001

To investigate the effect of long-term HFD in aging LEV rats, we measured food intake and body weight of eight HFD-fed and eight CD-fed male rats between 23 and 75 weeks of age. Food intake was almost equal in both groups but declined slightly with increasing age (Fig. 1a). The average body weight in the HFD group was 14% (*p* ≤ 0.01) higher than that in the CD group throughout the entire study. At the end of the study, the body weight difference between the groups was18% (*p* ≤ 0.001), indicating obesity in the HFD-fed rats (Fig. 1b). Fasting blood glucose, urea and total protein concentrations were similar in all animals, but the levels of serum LDL, HDL, and triglycerides were increased in obese animals compared to those in the control animals (Table 1). Creatine kinase levels were also significantly increased in obese animals. This enzyme plays a pivotal role in cellular energy homeostasis, particularly in tissues with highly dynamic energy demands such as muscles [17]. Therefore, we assessed different skeletal muscle parameters. The mean grip strength of the HFD group was significantly lower than that of the CD control group (Fig. 1c). MRI-based examination of the *musculus vastus lateralis* cross-sectional area showed a slight but significant decrease in the relative cross-sectional area in the HFD group, which was consistent with the results from the grip strength test (Fig. 1d, left panel). A postmortem histological analysis of the *musculus vastus lateralis* revealed almost equal numbers of muscle fibers in both groups (Fig. 1d, right panel).

**Table 1:** Blood chemistry in CD- or HFD-fed LEV rats

	CD	HFD
creatinine [mg/dl]	0.37 ± 0.03	0.4 ± 0.04
urea [mg/dl]	25.77 ± 1.27	25.16 ± 3.22
cholesterol [mg/dl]	83.37 ± 7.71	95.75 ± 14.93
LDL [mg/dl]	16.37 ± 2.46	19.5 ± 4*
HDL [mg/dl]	54.62 ± 5.87	60.12 ± 5.93*
triglycerides [mg/dl]	88.62 ± 10.37	151.12 ± 37.37*
glucose [mg/dl]	115.08 ± 7.83	115.67 ± 8.08
yGT [U/l]	2.25 ± 1.56	6.37 ± 2.56
Na [mmol/l]	145.67 ± 2.76	147.5 ± 2.65
albumin [g/dl]	4.33 ± 0.09	4.2 ± 0.2
total protein [g/dl]	6.6 ± 0.2	6.66 ± 0.13
Ca [mmol/l]	2.68 ± 0.07	2.75 ± 0.05
Mg [mmol/l]	0.55 ± 0.03	0.54 ± 0.03
creatine kinase [U/l]	226.62 ± 27.21	975.37 ± 323.21*

Values are given as the means ± SEM. Significant differences were analyzed using a two-way t-test and indicated by * for *p* ≤ 0.05 compared with CD-fed rats

### Long-term HFD leads to damage, inflammation and fibrosis in the kidneys of aging rats

**Fig. 2:**
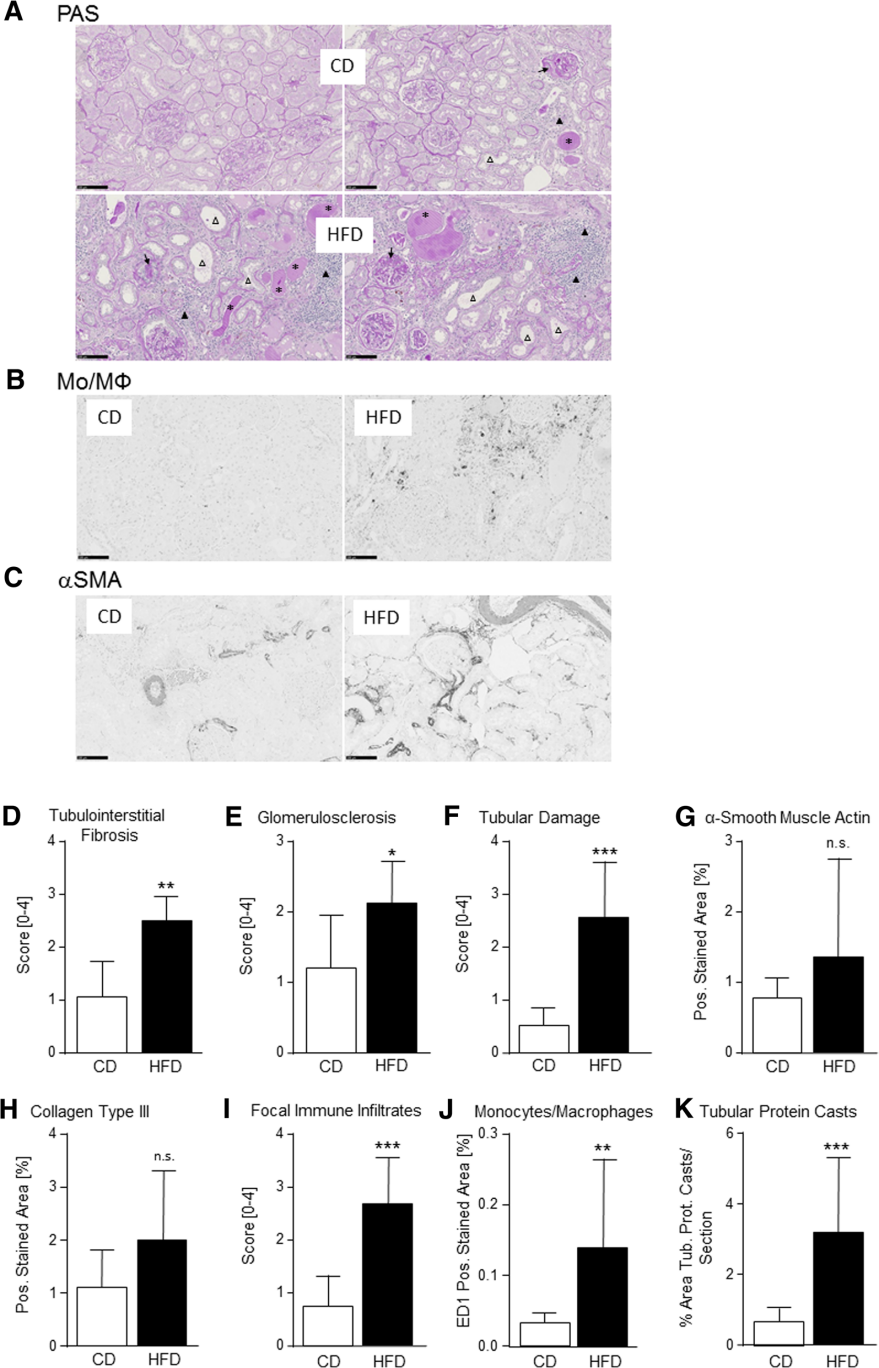
Renal inflammation and fibrosis in 18-month-old LEV rats. (**a**) PAS-stained renal tissues of 75-week-old rats show only subtle changes in those rats receiving a control diet (CD, upper two panels) compared to those fed a high-fat diet (HFD, lower two panels). In contrast to the CD, the HFD led to widespread tubular dilatation (open triangles), massive infiltration of mononuclear cells (filled triangles), and enhanced glomerulosclerosis (arrows) and tubular protein casts (stars). (**b**) The renal infiltration of ED1-positive monocytes/macrophages (Mo/MΦ) was significantly enhanced in rats fed a HFD. (**c**) Immunohistochemical staining of a-smooth muscle actin (aSMA) shows, next to the constitutive staining of smooth muscle cells, enhanced staining of profibrotic myofibroblasts in the tubulointerstitium of rats fed a HFD. (**d**)-(**k**) Quantification of the renal changes shows significantly enhanced tubulointerstitial fibrosis (**d**), glomerulosclerosis (**e**) and tubular damage (**f**) in the HFD-fed rats compared to CD-fed rats with a tendency for increased expression of aSMA (**g**) and type III collagen (**h**). The number of foci with renal immune infiltrates (**i**) and the number of infiltrating Mo/MΦ (**j**) increased significantly by HFD compared to the CD. K. HFD-fed rats showed a significantly enlarged area of tubular protein casts, pointing to enhanced proteinuria. Statistical significance (HFD vs. CD) was determined using two-way t-tests or Mann-Whitney-U test and is denoted by * for *p* ≤ 0.05, ** for *p* ≤ 0.01 or *** for *p* ≤ 0.001

To investigate the potential consequences of HFD-induced long-term obesity on kidney damage, inflammation and fibrosis, we first performed comprehensive analyses of PAS-stained renal sections (Fig. 2a). In the kidneys of 75-week-old rats with continuous HFD consumption, we detected a significant increase in tubulointerstitial fibrosis (Fig. 2a and d), glomerulosclerosis (Fig. 2a and e), general tubular damage (Fig. 2a and f) and the number of focal immune infiltrates (Fig. 2a and i). Quantification of the immunohistochemical staining of renal tissues for deposition of the extracellular matrix protein collagen type III and for the consistent tubulointerstitial expression of αSMA, a marker of profibrotic, activated myofibroblasts, yielded values tending to be increased in HFD-fed rats compared to those in CD-fed animals (Fig. 2c, g and h). Following quantification of immunohistochemical data, the kidneys of HFD-fed rats were further characterized by a significantly increased number of infiltrating monocytes/macrophages compared to those of CD-fed animals (Fig. 2b and j). Since urine samples were no longer available, we assessed a surrogate marker for proteinuria, the area of tubular protein casts in PAS-stained sections of each kidney. We observed a significant (4.6-fold) increase in tubular protein casts in HFD-fed LEV rats compared to the control group at week 75 (Fig. 2k).

### Long-term HFD elevates chemokine expression in the kidneys of aging rats

**Fig. 3:**
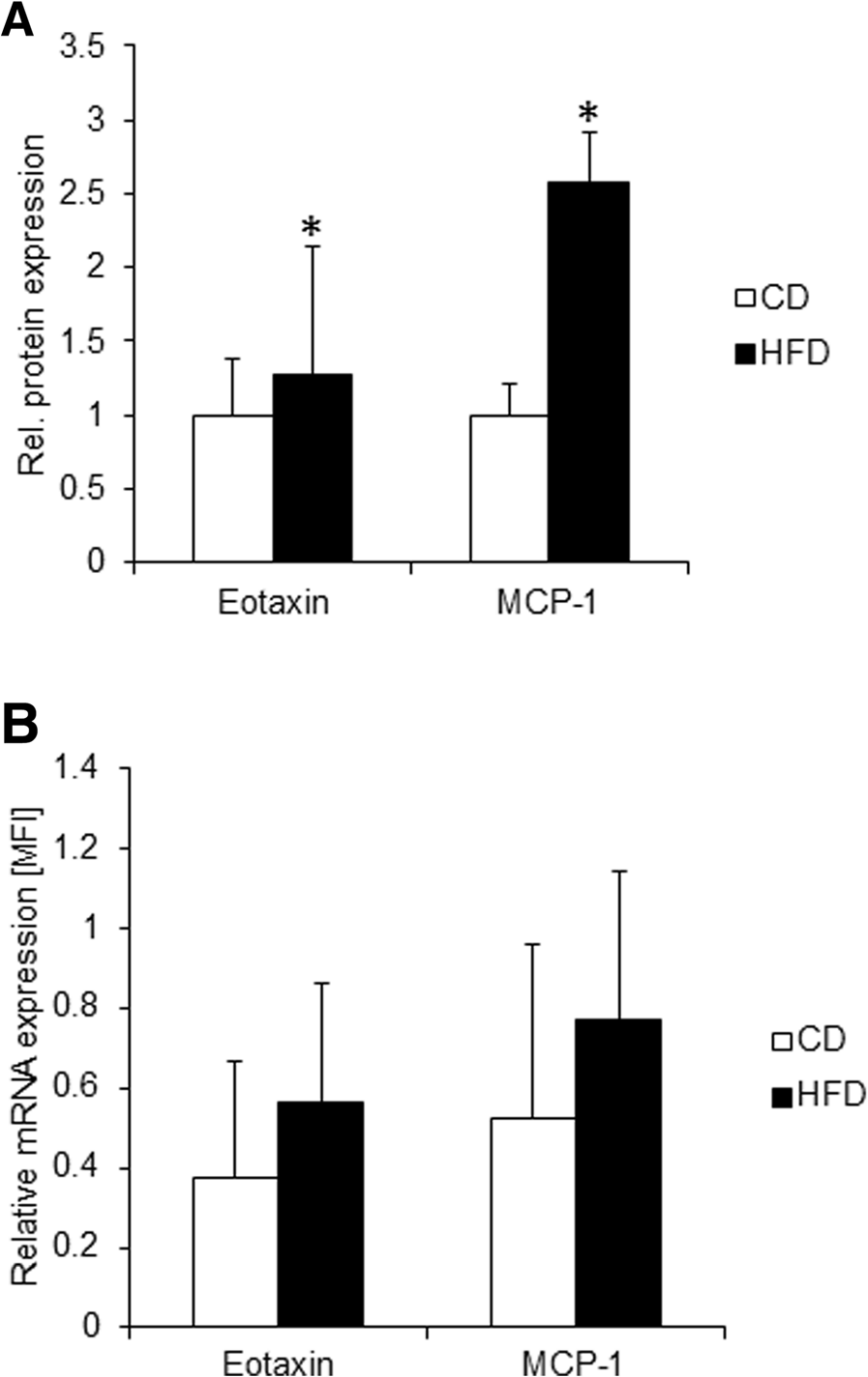
Chemokine expression in the kidneys of 18-month-old LEV rats. (**a**) The diagram shows the relative protein expression of eotaxin and MCP-1 in 1 ng of total kidney tissue extract from 18-month-old rats as the means ± SD of 8 CD-fed (open white bars) and 8 HFD-fed (solid black bars) male LEV rats. The mean eotaxin and MCP-1 levels in CD-fed rats were set to 1 for better comparison. Statistical significance (HFD vs. CD) was determined using two-way t-tests and is denoted by * for *p* ≤ 0.05. (**b**) The diagram shows the relative eotaxin and MCP-1 mRNA levels determined by QGP assay [MFI = mean fluorescence intensity] in kidneys from 18-month-old rats as the means ± SD of 8 CD-fed (open white bars) and 8 HFD-fed (solid black bars) male LEV rats. The differences between HFD- and CD-fed LEV rats were not significant as determined by two-way t-tests (*p* > 0.05)

Given that obesity-induced immune cell infiltration in the kidneys may be a result of the active production of chemokines, we used a preconfigured assay panel to determine the levels of 22 different chemokine proteins in equal amounts of total kidney extracts from all animals. Most proteins and their roles in renal inflammation have been recently discussed [18]. The levels of only two chemokines, MCP-1 (2.58-fold, *p* ≤ 0.05) and eotaxin (1.28-fold, *p* ≤ 0.05), were significantly increased in the kidneys from the HFD-fed LEV rats (Fig. 3a and Additional file 3). The relative mRNA expression of MCP-1 and eotaxin was also elevated in the HFD group, though it was insignificant and highly diverse among the animals (Fig. 3b).

## Discussion

Diet-induced obesity is well recognized as an important risk factor for renal impairment; however, the causal mechanisms remain elusive. The main findings here are that HFD-induced obesity promotes an inflammatory and fibrotic microenvironment in aging rat kidneys that is not only chemotactic for several types of immune cells but also associated with the development of renal lesions.

Experimental studies investigating obesity and its consequences are commonly performed in rats; however, the choice of strain can greatly influence the outcomes [16]. Moreover, a specific proportion of animals from the same strain is reported to become obese or hyperphagic [19]. Our observations suggest that the LEV strain is a reliable model for the experimental study of obesity. All 16 LEV rats in this study exhibited an increase in weight greater than 18% after 50 weeks of HFD consumption, and none of the animals were hyperphagic or showed atypical food consumption. The gain of 18% in body weight corresponds with the reported mean range of 10–30% weight increase in rats depending on strain and source of dietary fat [16]. Furthermore, we started HFD feeding of the LEV rats at the young age of 24 weeks, which was also reported to be most effective in inducing obesity in rats [20].

Animal models for obesity mainly use HFDs consisting of 30–78% of total energy intake from fat by adding a particular fat to the CD [21]. Not only the amount but the source of fat has also been reported to have an important impact on obesity [16]. Here, we used semipurified diets composed of identical fat types and sources with a defined macro- and micronutrient composition. Importantly, the components of the HFD and CD were identical, and the only difference between the CD and HFD in this study was the percentage of energy provided from plant-derived fat (Additional files 1 and 2). Thus, the phenotype reported here in the obese LEV rats is a direct consequence of the dietary fat content and obesity, resulting in elevated plasma levels of triglycerides, LDL, HDL and cholesterol.

HFD-induced obesity and excessive lipid accumulation have been suggested to impair skeletal muscle function in various rodent models [22, 23]. A previous study demonstrated that Sprague-Dawley rats, which were fed the same diet as the LEV rats in this study, are less susceptible to obesity but display a significant loss of muscle mass after long-term consumption of a HFD [24]. By comparison, obese LEV rats showed a minor decrease in muscle mass and function, as demonstrated by the grip strength test and muscle cross-sectional area or fiber quantity assessment (Figs. 1c and d). Therefore, obese LEV rats do not experience excessive degradation of muscle tissue and proteins, which could be a burden on the renal system.

One of the main findings of our study is that long-term obesity induces significant morphological changes in aging kidneys, which was characterized by enhanced renal inflammation, tubulointerstitial damage and fibrosis. This finding is in agreement with previous epidemiological human studies suggesting that obesity is a potential risk factor for the development of CKD independent of cardiovascular abnormalities, diabetes or metabolic syndrome [25–27]. Recent studies focused on renal energy metabolism using models of obesity reported direct effects from defective fatty acid oxidation or activation on the endocannabinoid system in tubular cells [28, 29]. Furthermore, a study using a genetic model of obesity associated with a mutation in the leptin receptor (*fa/fa* Zucker rats) reported a possible link between obesity and CKD [30]. In this context, the LEV rat model may be particularly relevant, as it allows the study of nutrition-mediated effects on kidney tissue remodeling as a direct consequence of a long-term HFD. Our model also closely reflects the conditions leading to the current human obesity epidemic resulting from the current industrialization of food systems [31, 32].

In addition to the cellular and structural changes in the kidneys of HFD-fed LEV rats, we observed a selective induction of two CC-type chemokine proteins, eotaxin and MCP-1, in crude kidney extracts. While both chemokines were significantly upregulated at the protein level in the kidneys of HFD-fed rats, this observation was less prominent at the mRNA level. This finding may suggest that eotaxin and MCP-1 genes are transcriptionally activated in a specific type or limited number of kidney cells, leading to a steady accumulation of encoded proteins. Another possible explanation is that eotaxin and MCP-1 expression is induced at the posttranscriptional level. Nevertheless, the elevated MCP-1 protein levels probably promote the infiltration of macrophages and monocytes, which express MCP-1 receptor CCR2 [11, 12]. Simultaneously, increased accumulation of eotaxin protein in the kidney is possibly a chemoattractant for eosinophils [13]. This could ultimately explain the significant morphological changes in the kidneys of HFD-fed aging LEV rats, which are characterized by enhanced inflammation, tubulointerstitial damage and fibrosis.

## Conclusions

Our data suggest that long-term HFD consumption and obesity induce an inflammatory microenvironment in the kidneys of aging LEV ratsthat is associated with glomerulosclerosis, tubular damage and an overabundance of macrophages and inflammatory markers. In particular, eotaxin and MCP-1 chemokines may be the key players in this scenario. Although the reported effects due to long-term HFD are relatively subtle, they may be sufficient to sensitize the kidneys to CKD.

## Methods

### Animal procedures

Twenty-four-week-old male LEV rats were fed either a HFD (43% energy from neutral fat, based on lard and corn oil) or a control diet (CD) (25% energy from neutral fat) until the age of 75 weeks (18 months). Compared with the CD, the HFD contained two-fold excess levels of all fatty acid species, and polysaccharides were reduced by 37% in compensation (Additional files 1 and 2). The HFD resulted in a 14% increase in metabolic energy compared with the CD. The concentrations of all other components, including proteins, were equal between the HFD and CD. All rats were given ad libitum access to water and food and were housed in groups of three rats per cage at a constant room temperature of 20 °C and on a 12-h light-dark cycle until the end of the experiment. The study included eight HFD-fed and eight CD-fed rats. Muscle strength was assessed using an automatic grip strength meter (Bioseb grip strength meter) according to the manufacturer’s instructions. The study protocol was approved by our local Committee on the Ethics of Animal Experiments.

### Blood samples

At the age of 75 weeks, rats were fasted overnight (16 h), and blood samples were drawn from the tail vein and transferred into heparin-coated vials. Plasma was immediately prepared and stored at − 20 °C pending further analysis. All parameters were measured at the central laboratory of the General Hospital Nuremberg using a COBAS C 702 analyzer (Roche Diagnostics, Mannheim, Germany).

### Magnetic resonance (MR) examination

LEV rats were anesthetized with an intraperitoneal injection of pentobarbital (Narcoren®, Merial, Hallbergmoos, Germany), and oxygen was delivered via a mask during subsequent examinations. MR imaging (MRI) was performed using Tesla clinical scanner (Magnetom, Siemens Healthcare, Erlangen, Germany). The cross-sectional area (CSA) was measured using T1-weighted, which were applied to image the quadriceps muscles of extended forelimbs. The variation coefficient in 15 consecutive measurements for the assessment of CSA was 1.77%, indicating high reproducibility.

### Histology and immunohistochemistry

A portion of the *musculus vastus lateralis* was partially fixed in 10% neutral buffered formaldehyde, embedded in paraffin, and cut into 5 μm sections. In hematoxylin-eosin (HE)-stained sections, 400 ± 10% myofibers from each animal were analyzed.

Renal tissue for light microscopy and immunohistochemistry was fixed in formalin and embedded in paraffin. Four-micron sections were stained with periodic acid-Schiff’s reagent (PAS) and counterstained with hematoxylin. In the PAS-stained sections, the percentage of focal or global glomerulosclerosis, the grade of tubular damage and tubulointerstitial fibrosis were determined as previously described [32–34]. The details are presented in additional file 4. The number of foci of immune infiltrates and the percentage of the total area of tubular protein casts was determined in the entire section area (mean average of 106.2 mm^2)^ following PAS staining.

For the renal immunohistochemistry, four-micron sections of formalin-fixed biopsy tissue were processed by an indirect immunoperoxidase technique and the positively stained tissue areas were quantified as previously described [34, 35]. More detailed information is provided in Additional file 4.

### MultiPlex immunoassay

A ProcartaPlex immunoassay (Thermo Fisher Scientific, Waltham, Massachusetts, USA) was used to assess the levels of 22 rat inflammatory markers (IL-1 alpha, G-CSF, IL-10, IL-17A, IL-1 beta, IL-6, TNF alpha, IL-4, GM-CSF, IFN gamma, IL-2, IL-5, IL-13, IL-12p70, eotaxin, GRO alpha, IP-10, MCP-1, MCP-3, MIP-1 alpha, MIP-2 and RANTES) in single muscle tissue samples. Sample preparation, assays and analyses were performed as described in the manufacturer’s instructions.

### QuantiGene Plex assay

Gene expression analysis was performed using a QuantiGene Plex assay (Affymetrix Inc., Santa Clara, CA), focusing on eotaxin, MCP-1 and the housekeeping genes PPIB and GAPDH. Tissue homogenates were transferred to a 96-well hybridization plate containing QuantiGene Plex probe and Luminex bead sets. The QuantiGene Plex assay was performed according to the manufacturer’s manual with all of the reagents and consumables supplied by the manufacturer (Thermo Fisher Scientific, Waltham, Massachusetts, USA).

### Statistical analysis

The data are presented as the means ± standard deviation (SD) in the diagrams or as the standard error of the mean (SEM) in the table. The parametric distribution of the data was assessed using D’Agostino-Pearson omnibus test. The statistical significance of the parametric data was analyzed using two-way t-tests (unpaired, unequal variance), and the Mann – Whitney U test was used to analyze the nonparametric data as specified in the figure legends. Differences with *p*-values ≤0.05 were considered statistically significant in all experiments. Significance levels were denoted by *p ≤ 0.05; **p ≤ 0.01; and ***p ≤ 0.001.

## Additional files

Additional file 1: CD (w/10% energy fom fat). (TIF 130 kb)

Additional file 2: HFD (w/45% energy fom fat). (TIF 130 kb)

Additional file 3: **Figure s3** Chemokine expression in the kidneys of 18-month-old LEV rats. (TIF 48 kb)

Additional file 4: Evaluation of histology and immunohistochemistry. (TIF 155 kb)

## Data Availability

The datasets used and/or analyzed during the current study are available from the corresponding author on reasonable request.
