# Supplementary figures and images for: High-fat diet-induced obesity causes an inflammatory microenvironment in the kidneys of aging Long-Evans rats
Source: J Inflamm (Lond). 2019 Jun 25;16:14. doi: 10.1186/s12950-019-0219-x (PMC6593534; doi:10.1186/s12950-019-0219-x)

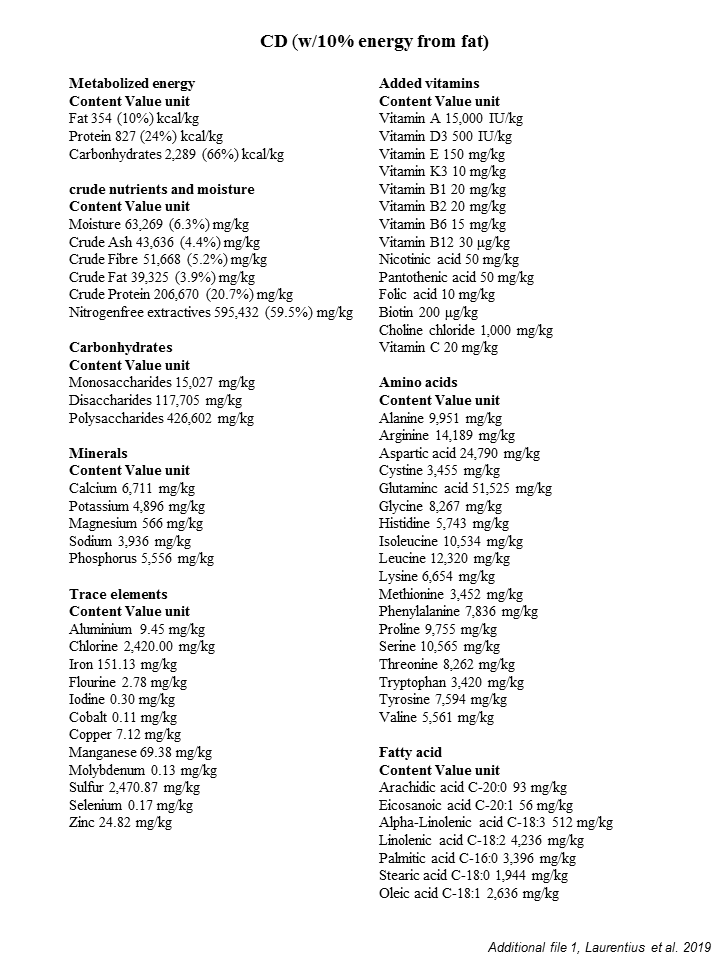

Supplement: Supplementary file 1 — CD (w/10% energy fom fat). (TIF 130 kb) [file 12950_2019_219_MOESM1_ESM.tif]

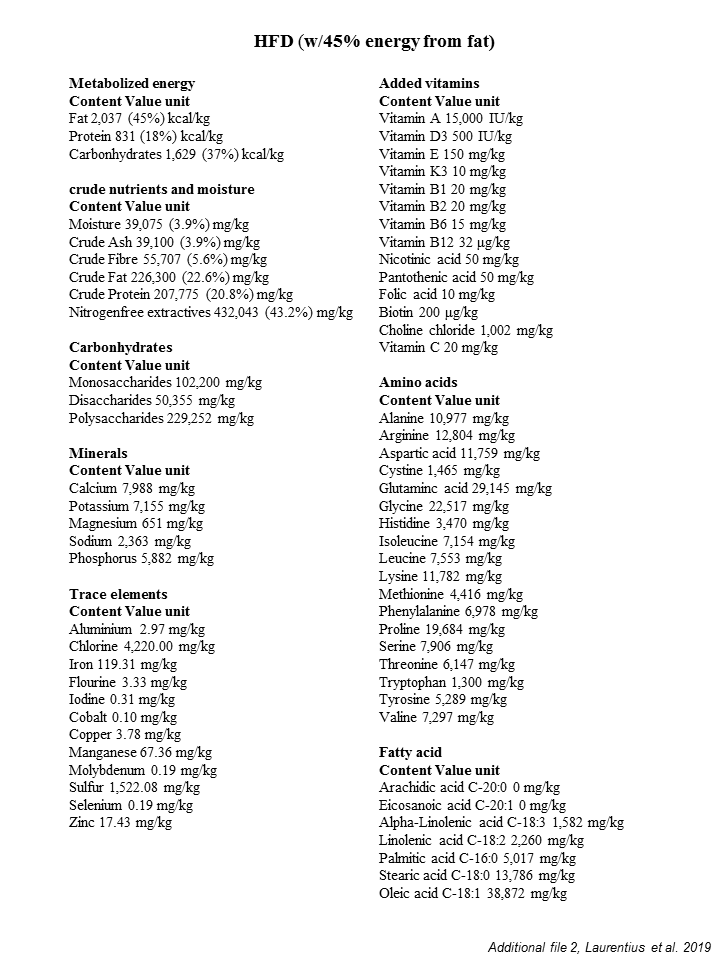

Supplement: Supplementary file 2 — HFD (w/45% energy fom fat). (TIF 130 kb) [file 12950_2019_219_MOESM2_ESM.tif]

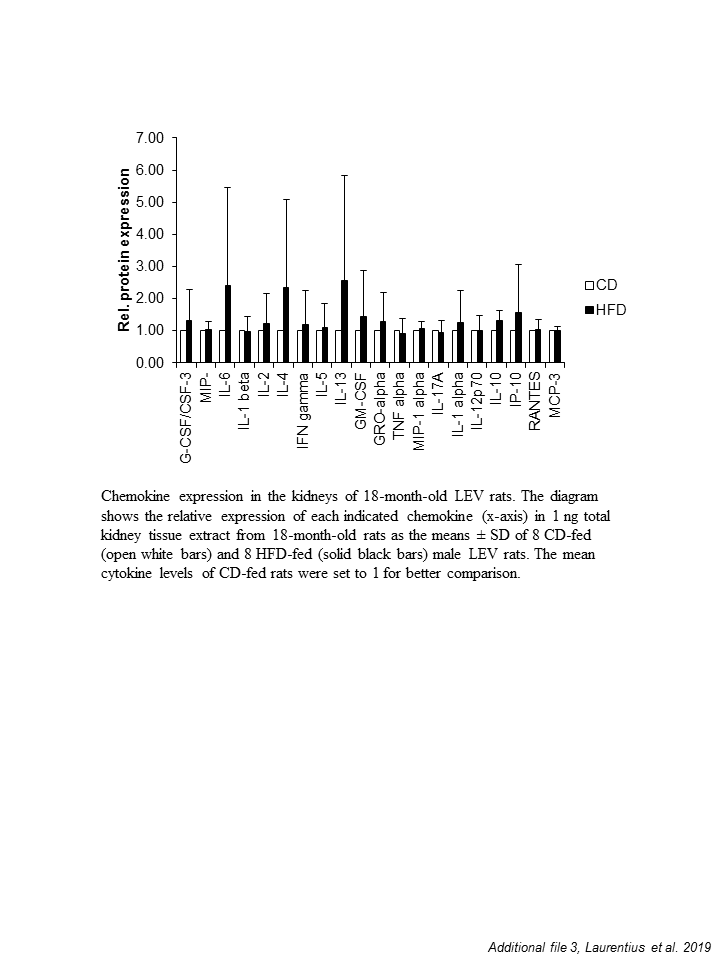

Supplement: Supplementary file 3 — Figure s3 Chemokine expression in the kidneys of 18-month-old LEV rats. (TIF 48 kb) [file 12950_2019_219_MOESM3_ESM.tif]

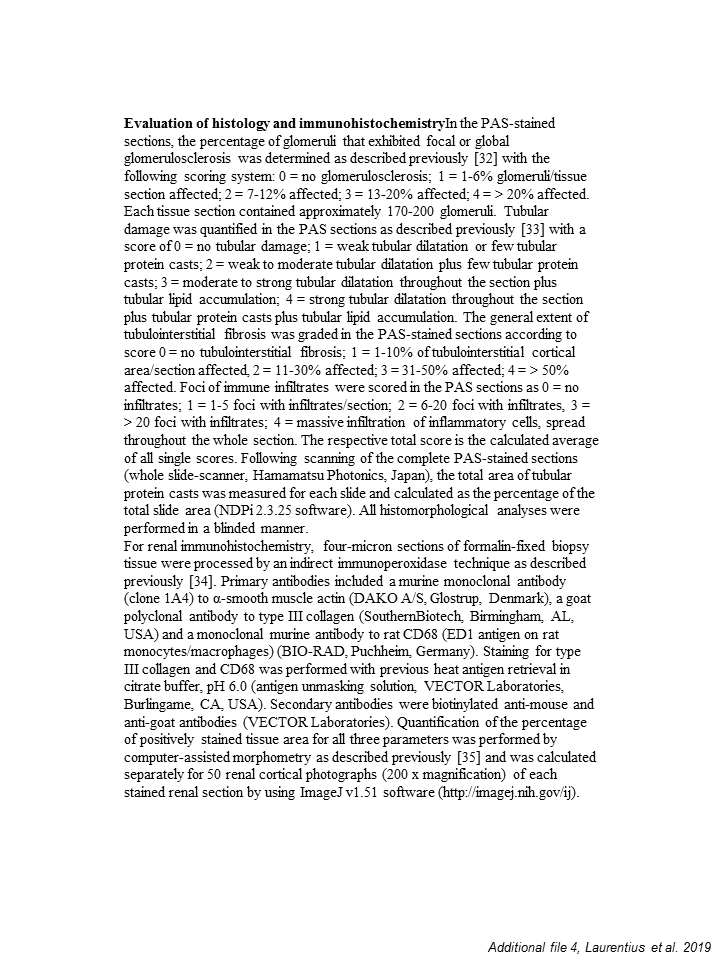

Supplement: Supplementary file 4 — Evaluation of histology and immunohistochemistry. (TIF 155 kb) [file 12950_2019_219_MOESM4_ESM.tif]
